# Toripalimab combined with concurrent platinum-based Chemoradiotherapy in patients with locally advanced cervical Cancer: an open-label, single-arm, phase II trial

**DOI:** 10.1186/s12885-022-09866-w

**Published:** 2022-07-19

**Authors:** Jie Chen, Chen Li, Yuanjie Cao, Li Zhu, Bailin Zhang, Jinqiang You, Hailing Hou, Jing Wang, Zhiyong Yuan

**Affiliations:** Department of Radiation Oncology, Tianjin Medical University Cancer Institute & Hospital, Key Laboratory of Cancer Prevention and Therapy, National Clinical Research Center for Cancer, Tianjin’s Clinical Research Center for Cancer, Tianjin, 300060 China

**Keywords:** Cervical cancer, Chemoradiotherapy, Immunotherapy, Immune checkpoint inhibitor, PD-L1 inhibitor, Toripalimab

## Abstract

**Background:**

Concurrent chemoradiotherapy is currently the standard of care for patients with locally advanced cervical cancer. However, even with the application of modern radiotherapy techniques, a considerable number of patients still develop distant metastases. PD-L1 inhibitors show good efficacy in cervical cancer. This single-arm phase II study aims to explore the efficacy and tolerability of combining PD-L1 inhibitor with concurrent chemoradiotherapy in the treatment of locally advanced cervical cancer.

**Methods/design:**

The primary endpoint of the study was the objective response rate assessed according to RECIST v1.1 criteria. The inclusion criteria were previously untreated patients aged 18–75 years with stage III-IVA (FIGO 2018 staging system) locally advanced cervical cancer. During concurrent chemoradiotherapy and consolidation chemotherapy, the enrolled patients will receive toripalimab (240 mg) every 3 weeks. After consolidation chemotherapy, the enrolled patients will be treated with toripalimab (240 mg) once every 6 weeks until the whole treatment cycle reaches 1 year. Intensity modulated radiotherapy was used for external beam radiation, and high-dose rate brachytherapy was delivered under image-guidance. Weekly DDP (40 mg/m^2^) was given concurrently with radiotherapy while 6 cycles of consolidated chemotherapy (paclitaxel plus DDP) were given after radiotherapy every three weeks. ﻿Secondary objectives included safety and tolerability, toxicity profile, progression-free survival, and overall survival.

**Discussion:**

PD-L1 inhibitor has shown good efficacy in recurrent/metastatic cervical cancer. However, there is still a lack of evidence about its combination with concurrent chemoradiotherapy in the treatment of locally advanced cervical cancer. The purpose of this study is to explore the efficacy and tolerance of this combination therapy, so as to lay the foundation for the future phase III randomized study.

**Trial registration:**

﻿clinicaltrials.govNCT05084677. ﻿Retrospectively registered on Octorber 07, 2021.

## Background

Cervical cancer is a common malignant tumor for female worldwide. According to the latest epidemiological survey by Globocan, the incidence and mortality rates of cervical cancer rank third among all female malignancies [1]. As a developing country, the incidence and mortality rates of cervical cancer are still relatively high in China, and there is still ﻿a substantial increase in cervical cancer incidence in contrast to the decreasing incidence trends in developed countries. ﻿In China, an estimated 98,900 new cases and 30,500 cervical-cancer related deaths happened in 2015 [2].

Concurrent chemoradiotherapy (CCRT) is currently the standard treatment for locally advanced cervical cancer (LACC). According to a meta-analysis of 13 studies, the addition of concurrent chemotherapy to radiotherapy alone improved the 5-year overall survival (OS) rate by 6% [3]. In recent years, with the development of radiotherapy and imaging techniques, the locoregional control of LACC has been greatly improved by using new technologies such as three-dimensional conformal radiotherapy (3DCRT), intensity-modulated radiotherapy (IMRT), image-guided adaptive brachytherapy (IGABT), and interstitial brachytherapy (ISBT). Distant metastasis, on the other hand, has become the most common type of treatment failure, occurring in 24–30% at 5 years after chemoradiation and brachytherapy [4–6]. Therefore, LACC patients may benefit from more intensified systematic treatment.

In recent years, immune checkpoint inhibitors targeted at ﻿programmed death-1(PD-1)/ ﻿programmed death-ligand 1 (PD-L1) pathway have shown antitumor activity in multiple tumor types [7, 8]. PD-1 is a receptor mainly expressed on activated T cells. By binding to its receptor, PD-L1 mainly expressed on tumor cells, the immune response is inhibited [9]. Considering the fact that ﻿persistent infection of high-risk human papillomavirus (HPV) is the main cause of cervical cancer, ﻿and that the presence of virus could lead to increased production of antigens ﻿identified as ﻿strong immune stimulants, evaluating immune checkpoint inhibition as a treatment strategy in cervical cancer is of great interest [10, 11]. ﻿Besides, in squamous cell carcinoma (SCC), a predominant histologic subtype accounting for approximately 80% of cervical cancer, the expression of PD-L1 is as high as 41–88%, which further ﻿provides a rationale to support the addition of immunotherapy in cervical cancer treatment [12–14]. In fact, there have been several prospective studies exploring the efficacy of PD-1 inhibitors in patients with recurrent/ metastatic cervical cancer [15–17]. Results showed that the overall objective response rate (ORR) was between 12.2 and 55.6%. Pembrolizumab was therefore approved by the US Food and Drug Administration for patients with PD-L1–positive LACC experiencing progression during or after chemotherapy.

Toripalimab is a recombinant, humanized IgG4 monoclonal antibody that prevents binding of PD-1 with PD-L1 and PD-L2. At present, it has received conditional approval in China for the treatment of unresectable or metastatic melanoma that has failed previous systemic therapy [18]. This study is a single arm prospective phase II clinical study aiming to explore the efficacy and tolerance of adding toripalimab simultaneously and subsequently to CCRT in patients with LACC.

The study started on January, 2021 and the duration of inclusion will be approximately 2 years.

## Methods/design

### Study design and objectives

**Fig. 1:**
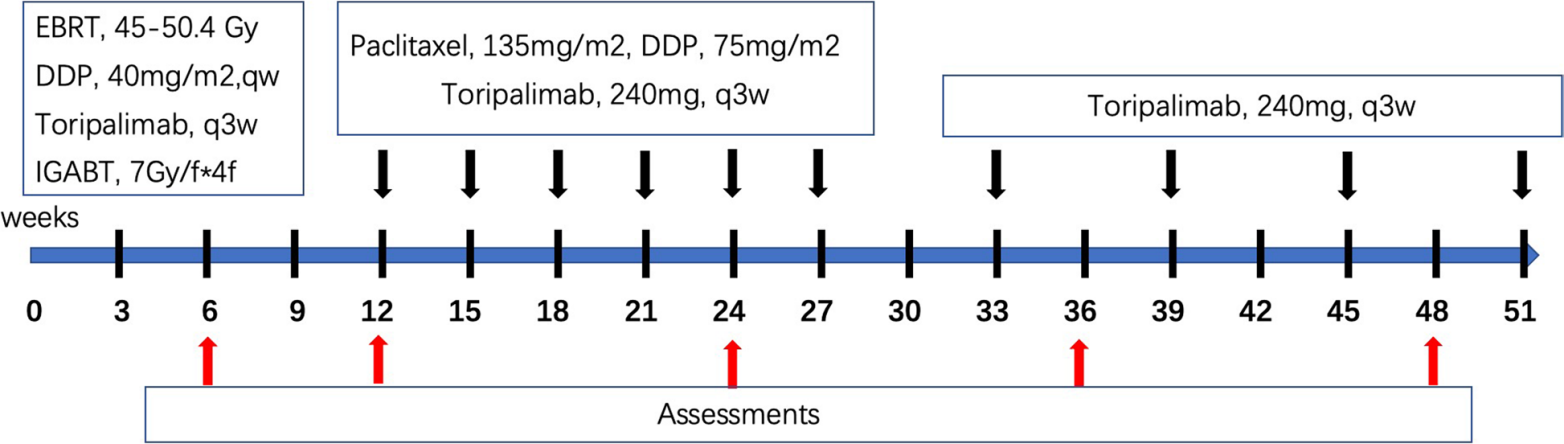
Schematic illustration of the therapy and evaluation procedures. Abbreviations: EBRT: external beam radiotherapy; DDP: cisplatin; IGABT: image-guided adaptive brachytherapy; QW: once per week; Q3W: once per 3 weeks

This study is a single-institution, open-label, single-arm, prospective phase II clinical trial. The enrolled patients will receive ﻿triple modality therapy comprised of radiotherapy, chemotherapy and immunotherapy, and the duration of immunotherapy will last for 1 year. ﻿A detailed overview can be found in the study scheme (Fig. 1). The aim of this trial is to investigate the efficacy of the addition of toripalimab to the standard CCRT treatment in LACC. The primary endpoint is the ORR. Secondary endpoints are the 2-year and 3-year progression free survival (PFS) rates, 3-year OS rate and the safety of the treatment protocol evaluated by NCI CTCAE V5.0.

#### Key eligibility criteria

The included patients are treatment-naive and diagnosed with histopathologically confirmed stage III-IVA (2018 FIOG staging system) LACC. Besides, patients with an Eastern Cooperative Oncology Group (ECOG) performance status 0 to 1, adequate organ function, no history of active severe comorbidities, and no history of active autoimmune disease are eligible for this trial (detailed key inclusion and exclusion criteria are listed in Table 1). Whether patients received prophylactic HPV vaccines or not does not affect their enrollment, whereas patients received therapeutic HPV vaccines after diagnosis of LACC were excluded from the enrollment.

**Table 1:** Key eligible criteria of the trial

Key inclusion and exclusion criteria	
Inclusion criteria	Exclusion criteria
Age between 18 and 75	Uncontrolled active infection
Untreated patients with pathologically proven stage III-IVA (2018 FIGO staging system) LACC	Prior malignancies (other than curable non-melanoma skin cancer) within 5 years
ECOG Performance Status of 0–1	Uncontrolled hypertension or diabetes, severe comorbidities such as cerebral embolism, cerebral hemorrhage, myocardial infarction or serious arrhythmia within 6 months
Adequate ﻿hematological, renal and hepatic functions: ﻿a. Hemoglobin > 8.0 g/dl b. Neutrophils > 2 × 10^9^/L; Leukocytes > 4 × 10^9^/L c. Platelets > ﻿100 × 10^9^/L g. Serum ALT/AST ≤ ﻿2.5× UNL h. Serum Total bilirubin ≤1.5× UNL d. Serum urea nitrogen ≤1.5 × UNL e. Serum creatinine (Cr) ≤ 1.5 × UNL	Patients who need to receive systemic corticosteroids (dose ≥10 mg prednisone qd) or other immunosuppressants within 14 days before enrollment or during the study
Life expectancy > 6 months	Vaccination of live attenuated vaccine within 30 days before enrollment, or plan to receive live attenuated vaccine during the study; Received therapeutic HPV vaccines after diagnosis of LACC
Eligible for CCRT assessed by principal investigator	Previous organ transplantation or HIV patients
No obvious active bleeding	Severe uncontrolled mental illness
Negative pregnancy test for patients at childbearing age, and voluntarily take effective and reliable contraceptive measures during the trial	Active acute or chronic viral hepatitis B or C. HBV DNA > 2000 IU/ml or 104 copies/ml; HCV RNA > 103 copies/ml
Written informed consent must be available before study registration	Patients with recurrent or distant metastatic disease
	Allergic to macromolecular proteins /monoclonal antibodies, or to any components of chemotherapeutic drugs used in the trial
	Active autoimmune diseases requiring systemic treatment or other diseases requiring long-term use of substantial glucocorticoids or other immunosuppressants

*Abbreviations*: *FIGO* International Federation of Gynecology and Obstetrics, *LACC* locally advanced cervical cancer, *ECOG* Eastern Cooperative Oncology Group, *ULN* Upper Limit of Normal, *CCRT* Concurrent Chemoradiotherapy

#### Pre-treatment evaluation

Detailed items of pre-treatment evaluations are shown in Table 2. Imaging evaluations of the tumor included enhanced MRI of the pelvis, ultrasonography of the abdomen and ultrasonography of cervical and supraclavicular lymph nodes. PET-CT is recommended for patients with multiple lymph node metastasis or suspected distal metastasis. Bone scan was performed for patients with bone pain or abnormally elevated serum alkaline phosphatase, and cranial MRI was performed when clinically indicated.

**Table 2:** Evaluation items before, during and after treatment

Eligibility	﻿Inclusion in trial	﻿Planning of RT (week ^− 2^)	﻿CCRT (week 0–8)	﻿End of EBRT (week 5)	﻿Follow-up 1 (month 3)	﻿Follow-up 2 (month 6)
﻿Written informed consent	✓					
Demographic information	✓					
﻿Medical History (incl. Histology)	✓				✓	✓
Physical Examination	✓			✓	✓	✓
Staging imaging	✓			✓	✓	✓
Pathological results	✓					
HPV testing	✓			✓		
Blood samples (Blood routine, hepatic and renal function, SCC, etc.)	✓		✓	✓	✓	✓
Planning CT		✓				
﻿Image-guidance during RT			✓			
﻿Toxicity (NCI CTC AE V5.0)			✓	✓	✓	✓

*Abbreviations*: *RT* Radiotherapy, *CCRT* Concurrent chemoradiotherapy, *EBRT* External beam radiotherapy, *SCC* Squamous cell carcinoma-antigen, *CT* Computed tomography

### Statistical analysis & sample size considerations

The ORR of patients with LACC after receiving (chemo) radiotherapy is approximately 65% according to previous literature [19]. We assumed that the addition of PD-1 antibodies to traditional treatment would yield an elevation of ORR to 80%. Using the optimal design principle of Simon’s Phase II ﻿two-stage minmax design, setting unilateral α = 0.025, β = 0.2, the optimal sample size required is 87 cases [20]. 27 patients were enrolled in the first stage. If less than 19 patients achieved complete response (CR) or partial response (PR), the trial will be terminated. If more than 19 patients achieved CR or PR, the trial would proceed to the second phase. In this phase, 60 additional patients will be included. Finally, if 64 patients reached CR or PR, it was confirmed that the treatment scheme was effective. Considering the shedding rate of 10%, 96 cases were needed.

### Radiotherapy

#### External beam radiotherapy (EBRT)

##### Planning CT

All patients will be positioned in prone position. In order to reduce the irradiation to bowels and bladder, all patients need to fast for at least 4 hours before simulation and before each radiotherapy fraction. Patients should take 500 ml water containing contrast agent orally 1 h before positioning to fill the bladder and to visualize the small intestine (contrast agent is omitted before daily irradiation). The patient is lying prone on a belly board, with both hands stretched forward and fixed by a thermoplastic body film. A contrast enhanced CT of the pelvis will be scanned and the slice thickness is set at 5 mm.

##### Contouring

The gross tumor volume (GTV) is defined as the ﻿macroscopic primary tumor. The GTV will be comprehensively determined by all available imaging resources (physical examination, colposcopy, CT- abdomen/pelvis, MRI-pelvis, PET-CT, etc.).

The metastatic regional node (GTVnd) is defined as any lymph node that can be diagnosed as or highly suspected of metastasis using all available imaging methods.

﻿The clinical target volume (CTV) includes the primary cervical tumor, cervix, uterus, upper half vagina, parametria, and pelvic lymph node regions (obturator, internal, external, common iliac and pre-sacral). Upper half vagina should be included in CTV for patients with no or minimal vaginal extension, upper two-thirds vagina for patients with upper vaginal involvement and the entire vagina for patients with extensive vaginal involvement. ﻿For patients with extensive pelvic node involvement /common iliac/ para-aortic lymph node involvement, CTV including the para-aortic lymph node up to the level of the renal vessels is recommended.

The planning metastatic regional node volume (PGTVnd) is defined as an expansion of 7 mm of the GTVnd, and the planning target volume (PTV) is derived from CTV plus a uniform 7 mm margin. Rectum, colon, small intestine, bladder, both femoral heads and pelvic bone will be contoured as organ at risk (OAR).

##### Dose prescription and irradiation

For patients without clinical positive lymph nodes, the prescription to 95% PTV is 45.0–50.4 Gy in 25–28 fractions, 1.8 Gy per fraction, 1 fraction per day, 5 days per week. For patients with clinical positive or highly-suspected positive lymph nodes, simultaneous integrated boost (SIB) will be used. The prescription to 95% PTV is still 45.0–50.4 Gy in 25–28 fractions, 1.8 Gy/fraction, 1 fraction/day, 5 days/week, while 95% PGTVnd will be boosted to 53.50–59.92 Gy simultaneously in 25–28 fractions, 2.14 Gy/fraction, 1 fraction/day, 5 days/week. EBRT techniques include IMRT, volumetric arc therapy (VMAT) and Helical Tomotherapy (TOMO). Image-guided radiotherapy (IGRT) is realized by ﻿cone-beam computed tomography (CBCT). For patients receiving IMRT or VMAT, CBCT is performed daily in the first week and weekly afterwards. For patients treated by TOMO (TomoTherapy Inc., Madison WI) or Halcyon accelerator (﻿Varian Medical Systems, Palo Alto, CA, USA), CBCT is performed before each fraction. Intensity-modulated radiotherapy plans were generated by the Pinnacle (Philips, Eindhoven, The Netherlands) or ﻿Eclipse (Varian Medical Systems, Palo Alto, CA, USA) treatment planning system. Irradiation was delivered with 6-MV photon energy. Dose coverage required that 95% of PTVs / PGTVnds receive the prescribed dose.

#### Intracavitary brachytherapy (ICBT)

IGABT was performed using a high-dose rate (HDR) after- loading system (Flexitron, Elekta). Pelvic CT/MRI was scanned during each fraction and target structures (residual tumor; high-risk and intermediate-risk CTV; and organs at risk) were delineated on transverse section images according to recommendations from GEC-ESTRO and International Committee on Radiation Units (ICRU) [21, 22]. ﻿Applicators were CT/MRI-compatible intracavitary ﻿intrauterine tandems and ovoids (Elekta). ﻿Interstitial needles were used for patients with more advanced disease at diagnosis or with extensive residual tumor in the middle of CCRT. Treatment planning was performed using OncCentra (Elekta). ﻿Brachytherapy dose was 28 Gy in four fractions of 7 Gy specified at 100% isodose around the high-risk CTV.

#### Dose constraints

﻿Small intestines, colon, rectum, bladder and femoral heads should be contoured on the simulation images as OARs. Bowels should be contoured at least 2 cm superior and inferior to PTV. OARs such as bladder and femoral heads should be delineated from top to bottom.

The volume of femoral head receiving 50Gy or more should not exceed 5% of the total volume (V50 < 5%). ﻿Other dose constraints to OARs include a maximal dose of 75 Gy to the ﻿maximally exposed 2 cm^3^ of rectum and sigmoid (D2cc sigmoid & D2cc rectum ≤75 Gy), and 85 Gy to the D2cc of the bladder (α/β = 3 Gy) in EqD2 (D2cc bladder ≤85 Gy).

### Chemotherapy

﻿During radiotherapy, cisplatin (40 mg/m^2^) will be used once a week for 4 to 6 weeks. After radiotherapy, six cycles of consolidated chemotherapy will be carried out as follows: ﻿paclitaxel 135 mg/m^2^ on day 1 and cisplatin 75 mg/m^2^ infused intravenously (i.v.) on day 2–4. ﻿Prophylactic antiemetic, acid-suppressive, liver-protection and nutritional supportive drugs will be routinely infused during every cycle of chemotherapy. Any anti-tumor cytotoxic drugs, targeted drugs or research drugs not included in this protocol will not be accepted.

### Immunotherapy

Toripalimab is given concurrently with and sequentially after CCRT. During CCRT, two cycles of 240 mg toripalimab are given intravenously once every three weeks. After CCRT, six cycles of 240 mg toripalimab are given concurrently with consolidated chemotherapy once every three weeks. After that, 240 mg toripalimab will be given once every six weeks until the whole treatment period reached one year since the beginning.

### Toxicity & dose modifications

Toxicity will be evaluated using the International Common Terminology Criteria for Adverse Events of the National Cancer Institute (NCI CTCAE), Version 5 during and after treatment. For chemotherapeutic drugs, if grade IV neutropenia, thrombocytopenia, leucopenia or grade III neutropenia with fever occurs, the dose of next cycle will be reduced by 20%. If the hematological toxicity has not recovered to grade 0 ~ I before next cycle after dose-reduction, the chemotherapy will be suspended; if the hematological toxicity recovers to grade 0 ~ I, the chemotherapy will be given by the original dosage. If grade IV non-hematological toxicity occurs, chemotherapy will be discontinued. For any side effects occurring during and after chemoradiation, the principal investigator should determine whether it is immunotherapy-related adverse event (irAE). The principles for handling irAEs are shown in the following Table 3:

**Table 3:** Recommended dose adjustments for toripalimab

﻿NCI CTCAE 5.0	Grade	Management
Pneumonitis	II	Interrupt toripalimab until restore to grade 0–I
	III-IV or recurrent grade II	Discontinue toripalimab
Diarrhea and colitis	II-III	Interrupt toripalimab until restore to grade 0–I
	IV	Discontinue toripalimab
Hepatitis	II (3× UNL<ALT/AST<﻿5× UNL, or 1.5 × UNL<total bilirubin<﻿3× UNL)	Interrupt toripalimab until restore to grade 0–I
	III-IV (ALT/AST>﻿5× UNL, or total bilirubin>﻿3× UNL)	Discontinue toripalimab
Nephritis	Grade II-III elevated blood creatinine	Interrupt toripalimab until restore to grade 0–I
	Grade IV elevated blood creatinine	Discontinue toripalimab
Endocrine disease	Symptomatic grade II-III hypothyroidism Grade II-III hyperthyroidism Grade II-III hypophysitis Grade II adrenal insufficiency, Grade III hyperglycemia or type 1 diabetes	Interrupt toripalimab or hormone replacement therapy until restore to grade 0–I
	Grade IV hypothyroidism Grade IV hyperthyroidism Grade IV hypophysitis Grade III-IV adrenal insufficiency Grade IV hyperglycemia or type 1 diabetes	hormone replacement therapy
Skin reactions	Grade III skin rash	Interrupt toripalimab until restore to grade 0–I
	Grade IV skin rash, Stevens-Johnson syndrome or toxic epidermal necrolysis	Discontinue toripalimab
Thrombocytopenia	III	﻿Interrupt toripalimab until restore to grade 0–1I
	IV	Discontinue toripalimab
Others	Grade II-III elevated blood amylase or elevated lipase Grade II pancreatitis Grade II myocarditis Other grade II-III irAEs that occur for the first time	Interrupt toripalimab until restore to grade 0–I
	Grade IV elevated blood amylase or elevated lipase Grade III-IV pancreatitis Grade III-IV myocarditis Grade III-IV encephalitis Other grade IV irAEs that occur for the first time	Discontinue toripalimab
Recurrent or persistent adverse events	Recurrent grade III-IV (except for endocrine diseases) Grade II-III adverse events not recover to grade 0-I within 12 weeks after the last administration (except for endocrine diseases) Corticosteroids failed to drop to a prednisone equivalent dose of ≤10 mg/day within 12 weeks after the last dose	Discontinue toripalimab
Infusion reaction	II	Reduce the drip rate or suspend the infusion, medication could be resumed under close observation when the symptoms are relieved
	III-IV	Discontinue toripalimab

*Abbreviations*: *ALT* Alanine aminotransferase, *AST* Aspartate aminotransferase, *UNL* Upper normal limit*, irAE* Immunotherapy-related adverse event

### Assessment of efficacy & follow-up

Efficacy evaluation will be conducted during and after treatment. Detailed evaluation items are listed in Table 1. Mid-treatment evaluation is carried out at the end of EBRT, mainly including physical examination, pelvic MRI and HPV testing. The main purpose of this evaluation is to better determine the method of brachytherapy (intracavity or interstitial). Major efficacy evaluation will be conducted 1 month after chemoradiation (3 months since the beginning of radiotherapy), which includes comprehensive imaging examination and blood tests (Table 1). Such evaluation will also be conducted every 3 months within 2 years of treatment, every 6 months within 3–5 years, and yearly after 5 years of treatment. CR, PR, stable disease (SD) and progressive disease (PD) will be defined for the target and non-target lesions at each follow-up according to the RECIST 1.1 -criteria. Patients suspected of disease recurrence / metastasis by routine examination will receive further examinations (PET-CT, histological examination, etc.).

OS time is defined as the time interval between the diagnosis of the disease and death for any cause. PFS time is defined as the time interval between the diagnosis of the disease and progression at any site or death for any cause.

## Discussion

Considering the high expression of PD-L1 in cervical cancer compared with other cancers, the application prospect of PD-L1 inhibitor in cervical cancer is promising. In the recently published Keynote-826 study, adding ﻿pembrolizumab to ﻿platinum-based chemotherapy has improved both PFS and OS rates of patients with persistent, recurrent or metastatic cervical cancer [23]. Toripalimab also showed promising efficacy in melanoma, urothelial cancer, renal cell cancer, nasopharyngeal cancer and multiple solid tumors [18, 24–26]. Therefore, exploring the combination of PD-L1 inhibitors and CCRT in the treatment of LACC has become a hot spot in recent research. Several prospective phase II / III studies exploring this aspect are being carried out simultaneously with our research, among which Keynote-A18 (NCT04221945) is the largest ongoing randomized phase III clinical trial comparing CCRT with or without pembrolizumab for the treatment of LACC. In addition, several smaller-size randomized studies have investigated the application of PD-L1 inhibitors as maintenance treatment following CCRT in cervical cancer (NCT02635360, ﻿NCT03833479, [27]. Another single-arm phase I trial explored the safety and side effects of simultaneous toripalimab during CCRT in cervical cancer (NCT04368273). Compared with the above studies, patients enrolled in our study had more advanced disease and lower heterogeneity. Most of the staging criteria for the above studies were stage IB3-IVA, while the patients enrolled in our study were limited to stage III-IVA (2018 FIGO staging system). By doing so, we could provide more experience on the combination of immune checkpoint inhibitors with CCRT for patients with more advanced cervical cancer, and also lay a foundation for future randomized phase III trials.

Another highlight of this study is the application of advanced radiation and imaging techniques. All enrolled patients will receive intensity-modulated EBRT and IGABT. For patients with positive lymph nodes, we will further apply SIB-IMRT to give simultaneous boost to metastatic lymph nodes. At present, the experience of applying SIB-IMRT in the treatment of LACC is relatively limited, and most of the published literature focused on the application of SIB-IMRT in the scenario of CCRT [28–30]. According to the two largest retrospective studies (including 74 and 75 patients respectively) so far, SIB-IMRT has shown promising oncological outcomes and low toxicities in patients with LACC. 75 patients from EMBRACE study received a median nodal boost dose of 62 Gy EQD2, only six patients recurred in boosted nodes with a median follow up of 30 months [30]. Jayatilakebanda, et al. delivered a total dose of 60 Gy in 25 fractions to radiologically abnormal pelvic nodes by SIB technique and found no increase in toxicity compared to node negative patients [29]. Based on these results, we adopted SIB-IMRT technique in our trial and designed a 53.50–59.92 Gy /25–28 fractions boost protocol. To the best of our knowledge, our research will be the first batch of clinical trials combining immunotherapy with advanced radiotherapy techniques in cervical cancer.

All enrolled patients will receive six cycles of adjuvant chemotherapy (paclitaxel plus cisplatin) after CCRT. The value of adding adjuvant chemotherapy (ACT) to CCRT in the treatment of LACC is controversial. Siriwan et al. has conducted a prospective randomized trial on this issue. The results showed that compared with CCRT group, significant decrease of systemic recurrences was observed in the ACT group [31]. In another randomized phase III clinical trial conducted by ﻿Duenas-Gonzalez, et al., ﻿gemcitabine plus cisplatin CCRT followed by BCT and adjuvant gemcitabine/cisplatin chemotherapy improved both OS (log-rank *P* = 0.0224; HR, 0.68; 95% CI, 0.49 to 0.95) and PFS ﻿(log-rank P = 0 .0227; hazard ratio [HR], 0.68; 95% CI, 0.49 to 0.95) compared with standard treatment [32]. In addition, further analysis of Duenas-Gonzalez’s study found that stage III/IVA patients with higher tumor load benefited more significantly [33]. These findings provide a basis for adding ACT to CCRT in the treatment of LACC.

To conclude, our study aims to explore the effectiveness and tolerance of the combination of CCRT and immunotherapy in the treatment of LACC while applying advanced radiotherapy and imaging techniques. The results of this study will provide valuable reference for future follow-up research.

## Data Availability

Not applicable – data collection is still ongoing.
